# The complete chloroplast genome sequence of *Rhododendron farrerae* Tate ex Sweet (Ericaceae)

**DOI:** 10.1080/23802359.2023.2294897

**Published:** 2024-01-04

**Authors:** Jianshuang Shen, Lu Huang

**Affiliations:** Hangzhou Animation & Game College, Hangzhou Vocational & Technical College, Hangzhou, China

**Keywords:** Chloroplast genome, Ericaceae, *Rhododendron farrerae*, Phylogenetic analysis

## Abstract

*Rhododendron farrerae* Tate ex Sweet 1831 is a species of ornamental plant found in southern China. In the present study, the complete chloroplast genome of *R. farrerae* was sequenced. The genome was 149,453 bp in length and lacked the typical quadripartite structure. The plastid genome contained 112 genes, including 74 protein-coding genes, 34 tRNA genes, and 4 rRNA genes. The overall GC content of the genome was 35.65%. Phylogenetic analysis of 25 chloroplast genomes revealed that *R. farrerae* was closely related to *Rhododendron huadingense*. This study could provide fundamental information for the distribution, utilization, and phylogenomics of *Rhododendron.*

## Introduction

*Rhododendron* plants are native to various regions around the world, including Asia, Europe, and North America, which are commonly found in both temperate and subtropical areas, with some species even thriving in alpine environments (Cribb 2007). The flowers of *Rhododendrons* typically have bell-shaped or funnel-shaped blooms, and many species produce clusters of flowers. In addition to their esthetic appeal, *Rhododendrons* also offer important ecological benefits. *Rhododendrons* are popular ornamental plants in gardens and landscapes due to their beautiful flowers and evergreen foliage. They are often used as hedging or screening plants, as well as in formal garden designs (Dampc and Luczkiewicz 2013). *Rhododendron farrerae* Tate ex Sweet 1831 is widespread in the shrublands and grasslands (Arthur 1955). It is an excellent material for bonsai and landscaping, with luxuriant, beautiful, germinating, pruning-resistant, and peculiar root-pile features (Ng and Corlett 2003). Currently, few studies have reported that *R. farrerae* produces abundant metabolic components in its leaves and flowers, especially highly active flavanones (Arthur 1955). The chloroplast (cp) genome can provide valuable information for species identification, genetics, and phylogeny owing to its conserved genome structure and high substitution rates compared to other organelles of the plant (Daniell et al. 2021; Li et al. 2021). To better understand the taxonomic and evolutionary relationships of *Rhododendron*, we assembled the complete cp genome of *R. farrerae* based on Illumina pair-end sequencing data.

## Materials and methods

**Figure 1.**
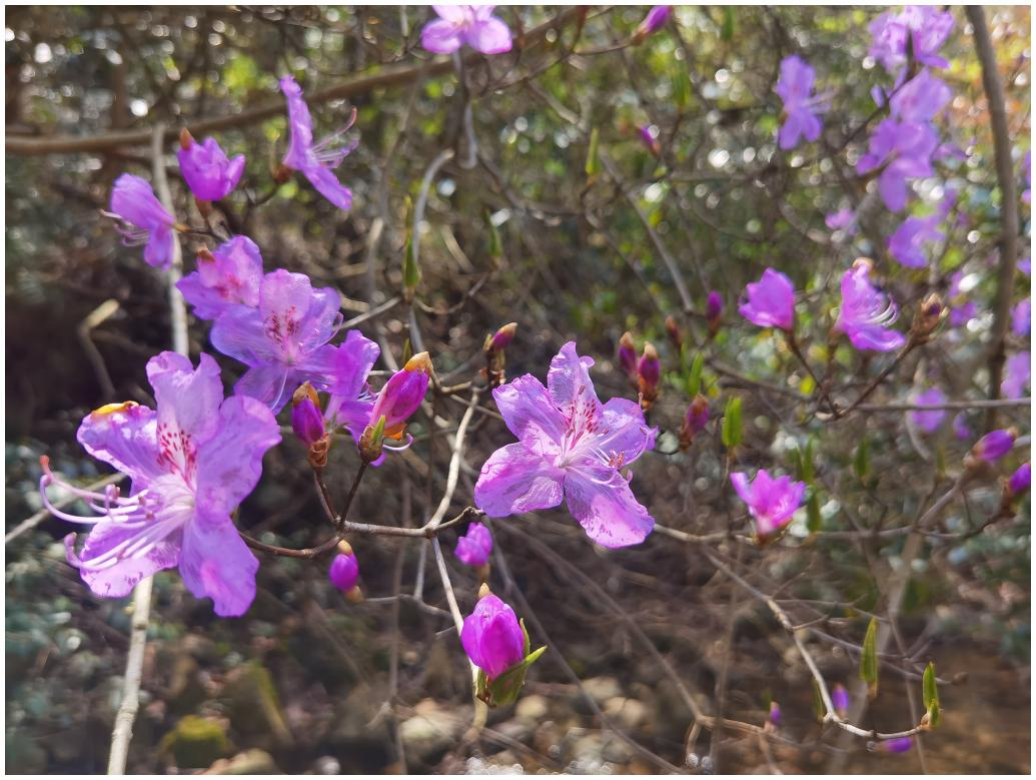
Reference image of *Rhododendron farrerae* plant. (taken by Jianshuang Shen, photographed in the mountain slope regions of the Zhejiang Province, China; the most characteristic feature of the specimen: Flowers 1-2 apical, flowers first and then leaves; corolla radial funnel-shaped, purple).

The samples of *R. farrerae* were grown in the mountain slope regions of the Zhejiang Province, China (Dongbai mountain, Zhuji:120°27′0″N, 29°30′0″E, Figure 1). Voucher specimens were deposited at the Institute of Botany (South China Botanical Garden Herbarium, Yinzhu Tang, and yinzhu@scbg.ac.cn) with the registration number 0450150. The leaf bud of *R. farrerae* were collected and frozen using liquid nitrogen at −80 °C. The modified cetyltrimethylammonium bromide (CTAB) method (Doyle 1987; Schenk et al. 2023) was used to extract total genomic DNA from *R. farrerae*. Fragmented 1 mg of purified DNA was used to set up 300 bp short-insert libraries. Paired-end DNA sequencing reads of 150 bp were generated using the Illumina NovaSeq 6000 platform (Illumina, San Diego, CA, USA), and fastp v0.20.0 and SPAdes v3.10.1 software were used to filter and assemble the genome, respectively (Shen et al. 2019; Zheng et al. 2020). *Rhododendron delavayi* complete chloroplast genome (cp genome) (GenBank accession number MN711645.4) was used as a reference for quality control after assembly. Prodigal v2.6.3 (https://www.github.com/hyattpd/Prodigal) was used to annotate the coding sequence of chloroplasts, HMMER v3.1b2 (http://www.hmmer.org/) was used to predict the rRNA genes, and ARAGORN v1.2.38 (http://www.ansikte.se/ARAGORN/) was used to predict the tRNA genes. A total of 17,858,393 paired-end reads were produced, with Q20 up to 96.67% and an average depth coverage of 2753 ×. The OGDRAW software (https://chlorobox.mpimp-golm.mpg.de/OGDraw.html) was used to generate the cp genome map. Finally, two taxa (*Actinidia deliciosa*, NC_026691.1, and *Actinidia chinensis*, NC_026690.1) were selected as outgroups and 22 complete chloroplast genomes of *Rhododendron* related to *R. farrerae* were selected to construct a phylogenetic tree. The sequences used in this study were downloaded from the NCBI GenBank. The complete cp genomes of the 24 species were aligned with the cp genome of *R. farrerae* from the same starting point using MAFFT v7.427 in auto mode. RAxML v8.2.10 (https://cme.h-its.org/exelixis/software.html) was used to build the phylogenetic tree using the GTRGAMMA model with 1000 bootstrap replicates.

## Results

**Figure 2.**
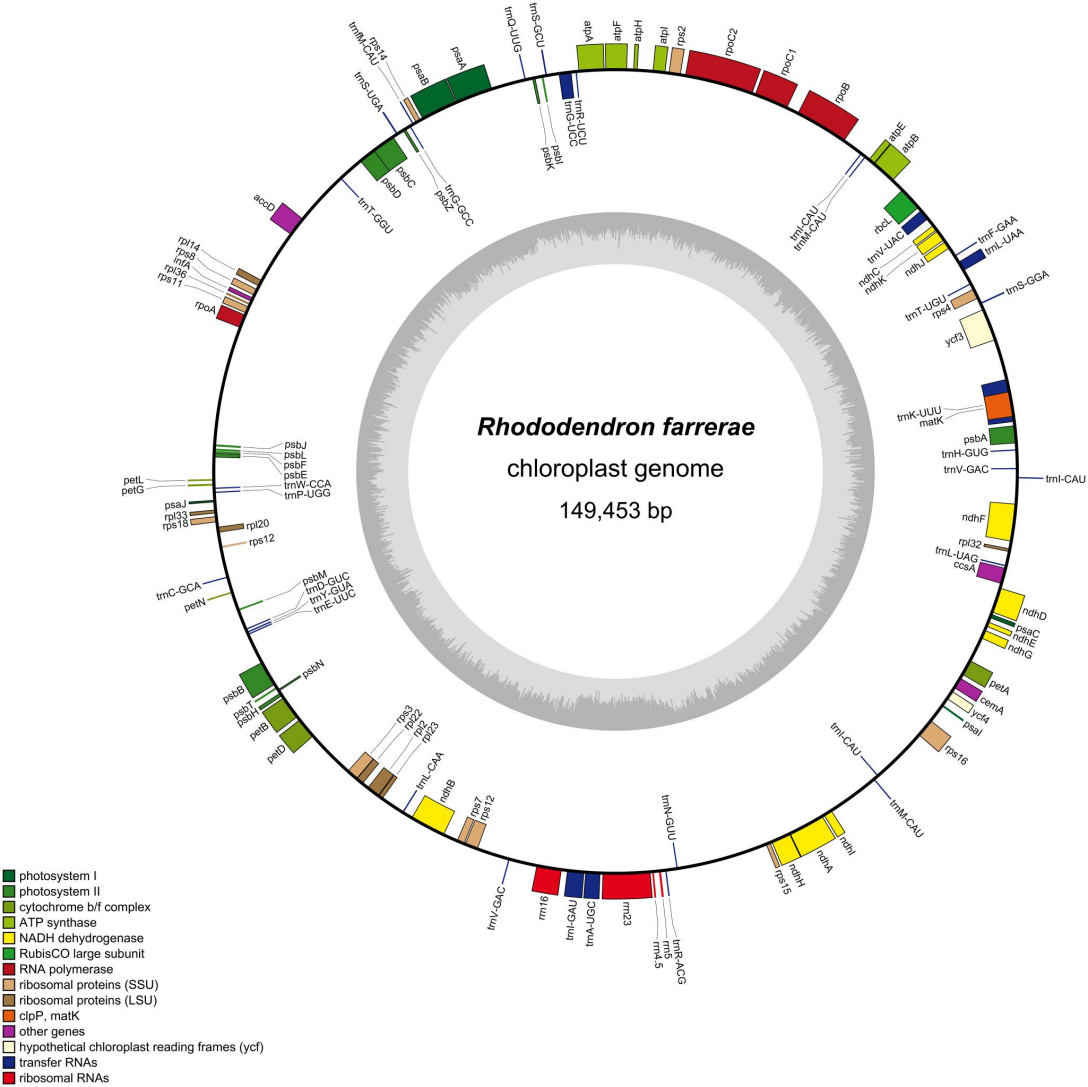
Gene map of the *Rhododendron farrerae* cp genome. Genes on the outside and inside of the circle are transcribed in the clockwise and counterclockwise directions, respectively. The dark and light gray bars in the inner circle denote G + C and A + T contents, respectively.

The cp genomes of *R. farrerae* (GenBank number: OQ980386; BioProject, SRA, and Bio-Sample numbers: PRJNA982689, SRR24901656, and SAMN35712182, respectively) were deposited in the NCBI database (BankIt2703782). The cp genome of *R. farrerae* was 149,453 bp in length, the quality control and read coverage depth map of the assembly of the cp genome had been shown in Figure S1 and Figure S2. The overall GC content of cp genome of *R. farrerae* is 35.65%. A total of 112 functional genes were identified, including 4 rRNA genes, 34 tRNA genes, and 74 protein-coding genes (Figure 2). Twelve genes contained one intron (*atp*F, *ndh*A, *ndh*B, *pet*B, *pet*D, *rps*16, *trn*A-UGC, *trn*G-UCC, *trn*I-GAU, *trn*K-UUU, *trn*L-UAA, and *trn*V-UAC), and two genes (*rps*12 and *ycf*3) had two. Moreover, both *trn*V-GAC and *trn*M-CAU had two copies, whereas *trn*I-CAU had three. Eight genes were difficult to annotate, including seven *cis*-splicing genes (*atp*F*, ndh*A*, ndh*B*, rps*16*, pet*B*, pet*D, and *ycf*3) and one *trans*-splicing gene (*rps*12) (Supplementary Figure S3).

**Figure 3.**
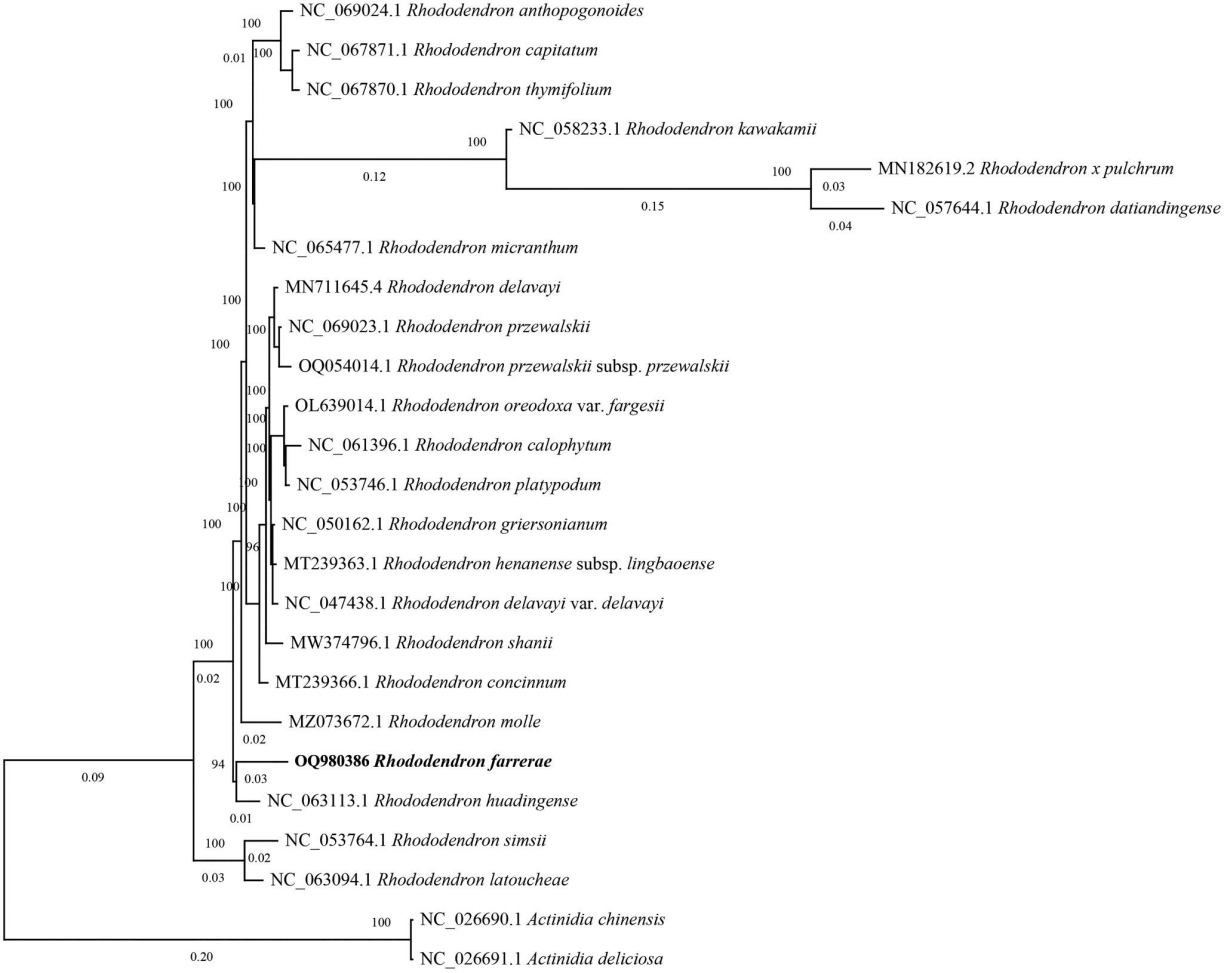
Phylogenetic tree of 25 species of *Rhododendron* obtained using GTRGAMMA model based on complete chloroplast genome. The complete chloroplast genomes of 25 species were used to construct a phylogenetic tree. The sequences used in this figure were downloaded from the NCBI GenBank. Two taxa (*Actinidia deliciosa* and *Actinidia chinensis*) are outgroups. Accession numbers are: *Rhododendron farrerae* (Accession number: OQ980386), *Rhododendron mole* (Accession number: MZ073672.1), *Rhododendron simsii* (Accession number: NC_053764.1), *Rhododendron × pulchrum* (Accession number: MN182619.2), *Rhododendron kawakamii* (Accession number: NC_058233.1), *Rhododendron henanense* subsp. Lingbaoense (Accession number: MT239363.1), *Rhododendron anthopogonoides* (Accession number: NC_069024.1), *Rhododendron datiandingense* (Accession number: NC_057644.1), *Rhododendron concinnum* (Accession number: MT239366.1), *Rhododendron micranthum* (Accession number: NC_065477.1), *Rhododendron przewalskii* (Accession number: NC_069023.1), *Rhododendron griersonianum* (Accession number: NC_050162.1), *Rhododendron shanii* (Accession number: MW374796.1), *Rhododendron delavayi* (Accession number: MN711645.4), *Rhododendron przewalskii* subsp. Przewalskii (Accession number: OQ054014.1), *Rhododendron thymifolium* (Accession number: NC_067870.1), *Rhododendron platypodum* (Accession number: NC_053746.1), *Rhododendron oreodoxa* var. fargesii (Accession number: OL639014.1), *Rhododendron capitatum* (Accession number: NC_067871.1), *Rhododendron calophytum* (Accession number: NC_061396.1), *Rhododendron huadingense* (Accession number: NC_063113.1), *Rhododendron delavayi* var. delavayi (Accession number: NC_047438.1), *Rhododendron latoucheae* (Accession number: NC_063094.1), *Actinidia deliciosa* (Accession number: NC_026691.1), *Actinidia chinensis* (Accession number: NC_026690.1).

The phylogenetic tree of *R. farrerae* with other members of *Rhododendron* was also explored, and the results showed that the cp genome of *R. farrerae* was closely related to *Rhododendron huadingense* (Figure 3 and Figure S4).

## Discussion and conclusion

The complete cp genome of *R. farrerae* was sequenced and analyzed. Our results indicate that the *R. farrerae* cp genome lacks the IRs, which was consistent with *R. pulchrum* verified using third-generation combined with second-generation sequencing methods (Shen et al. 2019, 2022). Recently study reported the cp genomes of the four *Rhododendron* species (*R. concinnum*, *R. henanense* subsp. *lingbaoense*, *R. micranthum*, and *R. simsii*) have a circular and quadripartite structure (Zhou et al. 2023). Further research on the cp genome structure of *Rhododendron* species is needed.

Chloroplast genome has been widely used for studying phylogenetic relationship. Previous phylogenetic studies of the genus *Rhododendron* clarified *R. farrerae* was closely related to *R. tashiroi* and *R. wadanum* based on chloroplast gene sequences (Kurashige et al. 2001; Kron and Powell 2009). With the increase of sequenced species, the phylogenetic relationship on the genus *Rhododendron* will be clear. In this study, the phylogenetic relationship of *R. farrerae* showed a consistent geographical distribution of the two wild *Rhododendron*, *R. huadingense* and *R. farrerae*, in the Zhejiang Province, China. This study provides fundamental information for the distribution, utilization, and phylogenomics of *Rhododendron.*

## Supplementary Material

Supplemental Material

## Data Availability

The genome sequence data that support the findings of this study are openly available in GenBank of NCBI at [https://www.ncbi.nlm.nih.gov/search/all/?term=OQ980386] under the accession no. OQ980386. The associated BioProject, SRA, and Bio-Sample numbers are PRJNA982689, SRR24901656, SAMN35712182 respectively.”
