# Supplementary material for: The complete chloroplast genome sequence of Rhododendron farrerae Tate ex Sweet (Ericaceae)
Source: Mitochondrial DNA B Resour. 2024 Jan 4;9(1):1–4. doi: 10.1080/23802359.2023.2294897 (PMC10769518; doi:10.1080/23802359.2023.2294897)
Supplement: Supplemental Material [file TMDN_A_2294897_SM4871.docx]

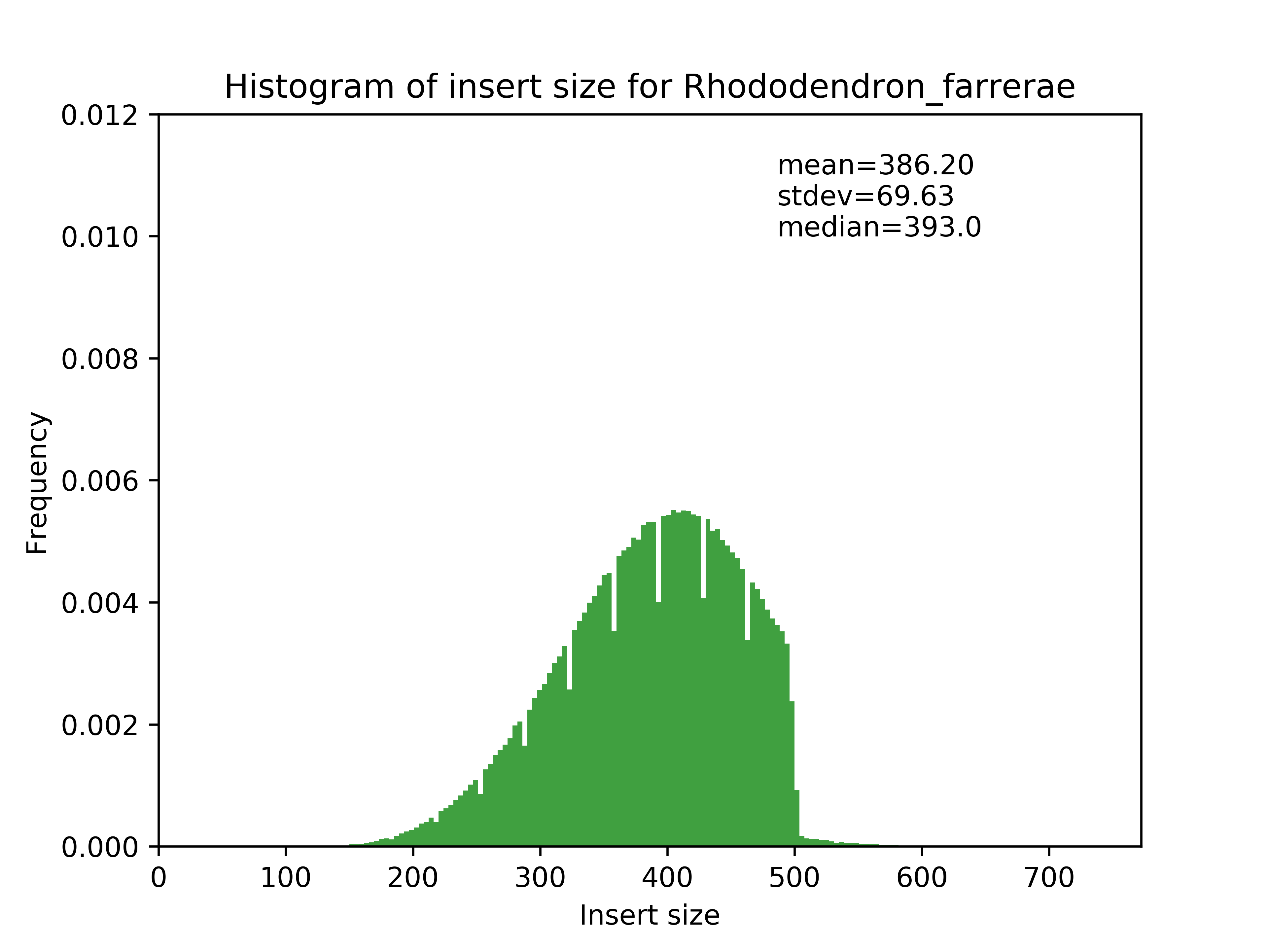


Supplement Figure S1 Quality control of the cp genome assembly of *Rhododendron farrerae*. (X-axis is the size of the insert and Y-axis is the frequency distribution).


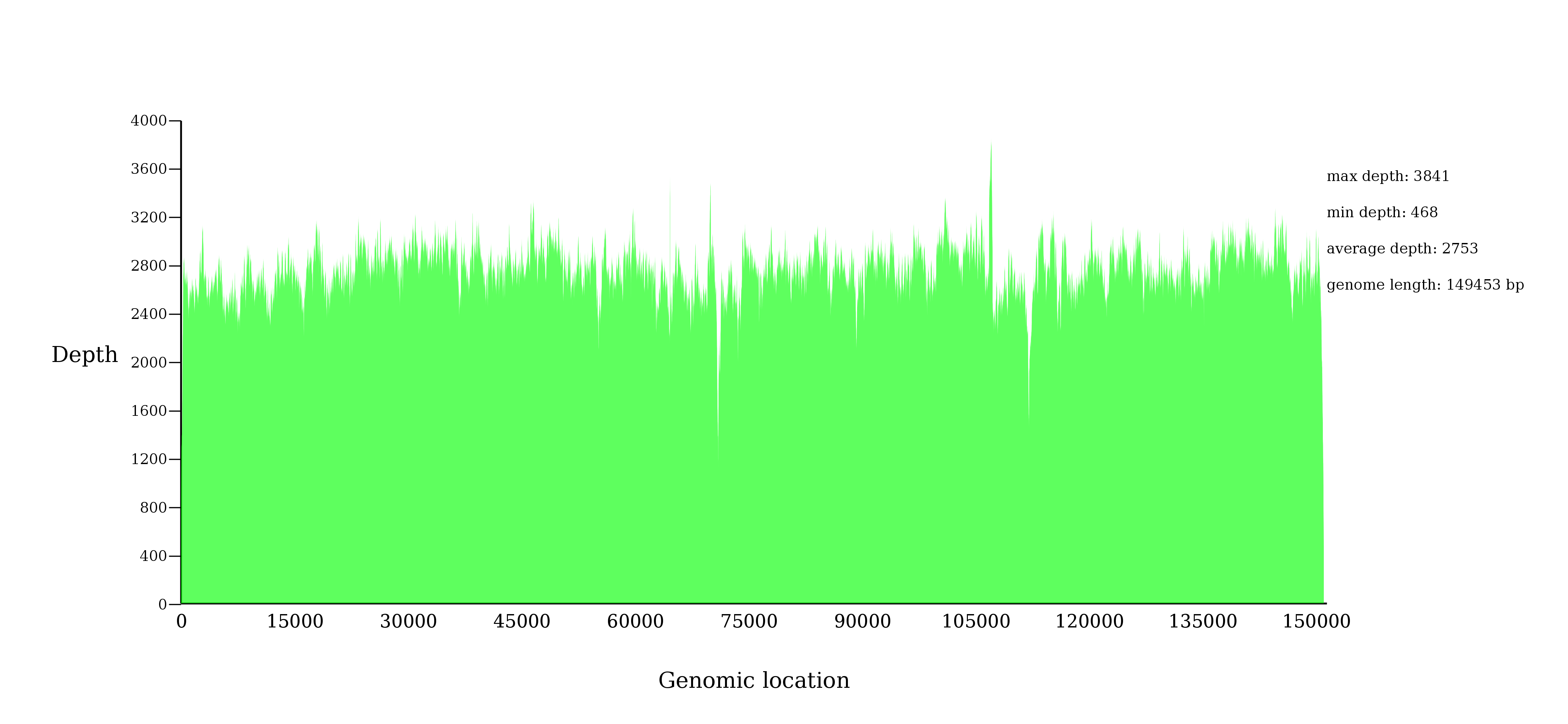


Supplement Figure S2 The read coverage depth map of the assembly of the cp genome of *Rhododendron farrerae*. (The figure means the coverage depth on each base of chloroplast genome of *Rhododendron farrerae*; X-axis indicates the location of bases in the genome; Y-axis indicates the coverage depth)


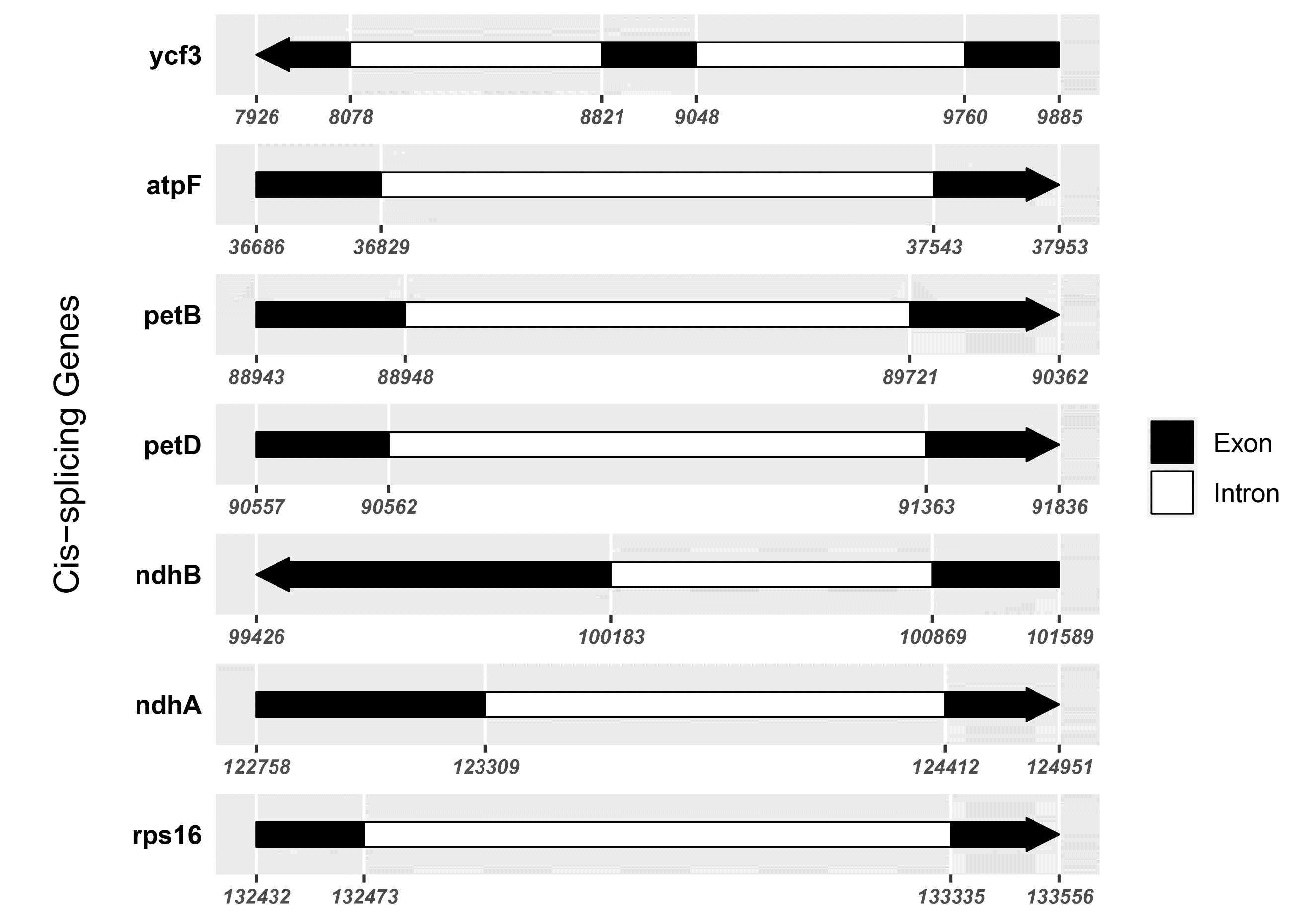

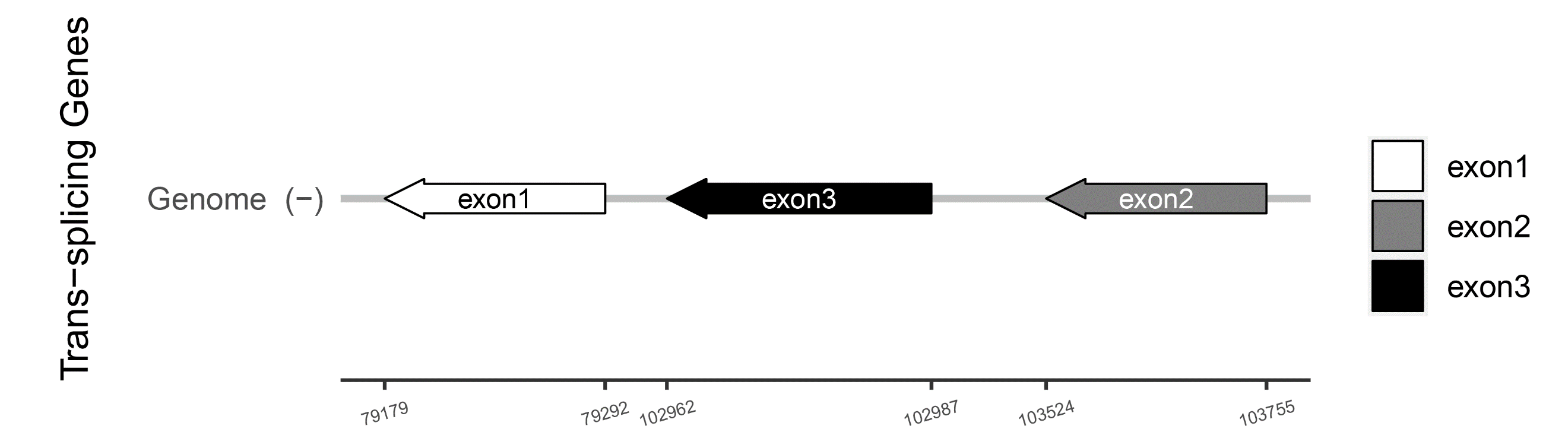


Supplement Figure S3 The structure of the 8 protein-coding trans- and cis-splicing genes annotated in Cp genome of *Rhododendron farrerae*. (including 7 cis-splicing genes and one trans-splicing gene *rps12*; The numbers in the picture represent the location in the Cp genome of *Rhododendron farrerae*)


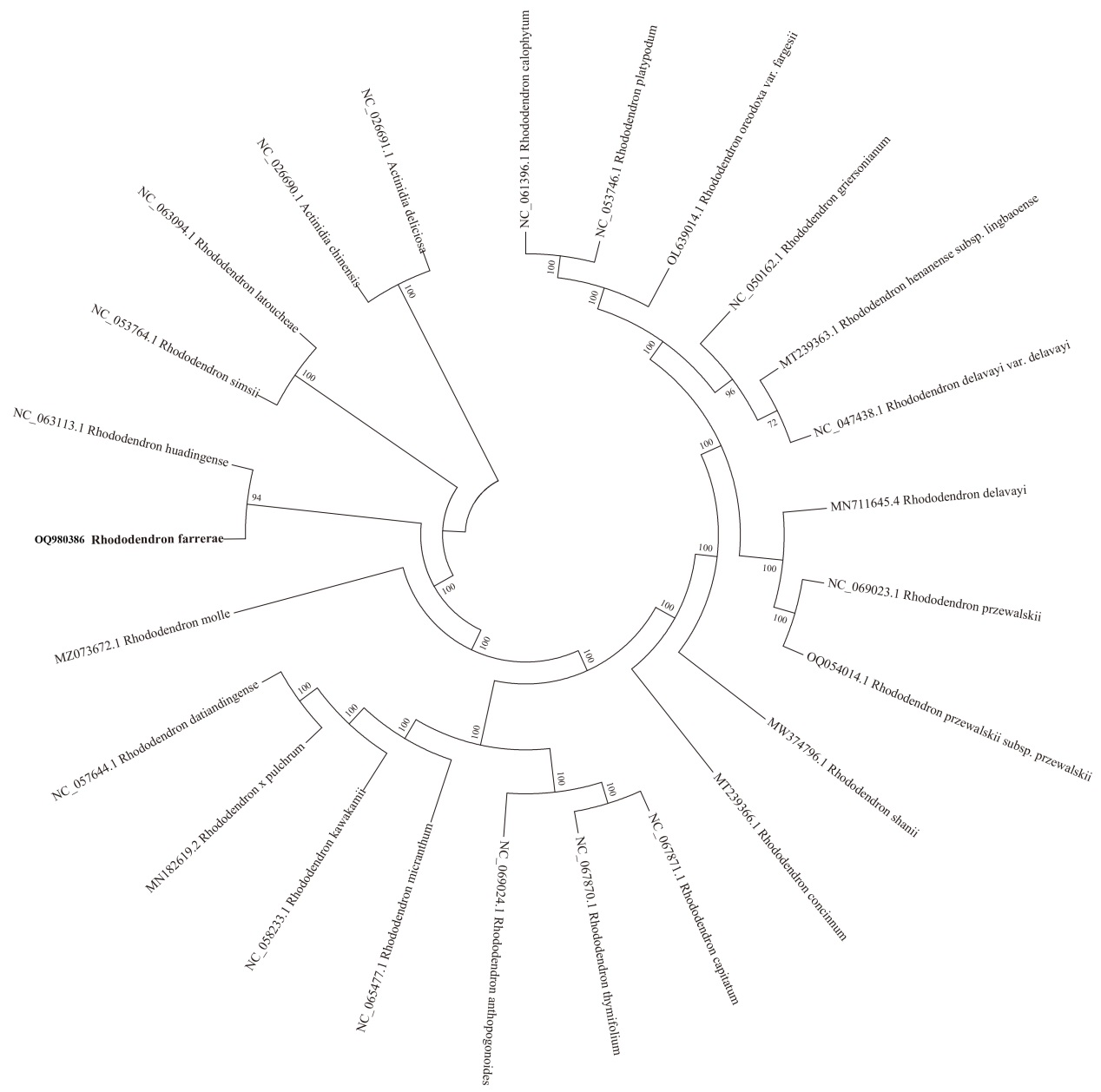


Supplement Figure S4 The cladogram tree of 25 species of *Rhododendron* obtained using GTRGAMMA model based on complete chloroplast genome. The complete chloroplast genomes of 25 species were used to construct a phylogenetic tree. The sequences used in this figure were downloaded from the NCBI GenBank. Two taxa (*Actinidia deliciosa* and *Actinidia chinensis*) are outgroups. Accession numbers are: *Rhododendron farrerae* (Accession number: OQ980386), *Rhododendron mole* (Accession number: MZ073672.1), *Rhododendron simsii* (Accession number: NC_053764.1), *Rhododendron × pulchrum* (Accession number: MN182619.2), *Rhododendron kawakamii* (Accession number: NC_058233.1), *Rhododendron henanense* subsp. Lingbaoense (Accession number: MT239363.1), *Rhododendron anthopogonoides* (Accession number: NC_069024.1), *Rhododendron datiandingense* (Accession number: NC_057644.1), *Rhododendron concinnum* (Accession number: MT239366.1), *Rhododendron micranthum* (Accession number: NC_065477.1), *Rhododendron przewalskii* (Accession number: NC_069023.1), *Rhododendron griersonianum* (Accession number: NC_050162.1), *Rhododendron shanii* (Accession number: MW374796.1), *Rhododendron delavayi* (Accession number: MN711645.4), *Rhododendron przewalskii* subsp. Przewalskii (Accession number: OQ054014.1), *Rhododendron thymifolium* (Accession number: NC_067870.1), *Rhododendron platypodum* (Accession number: NC_053746.1), *Rhododendron oreodoxa* var. fargesii (Accession number: OL639014.1), *Rhododendron capitatum* (Accession number: NC_067871.1), *Rhododendron calophytum* (Accession number: NC_061396.1), *Rhododendron huadingense* (Accession number: NC_063113.1), *Rhododendron delavayi* var. delavayi (Accession number: NC_047438.1), *Rhododendron latoucheae* (Accession number: NC_063094.1), *Actinidia deliciosa* (Accession number: NC_026691.1), *Actinidia chinensis* (Accession number: NC_026690.1).
